# Supplementary material for: Dietary Advanced Glycation End-Products (dAGEs) Intake and Bone Health: A Cross-Sectional Analysis in the Rotterdam Study
Source: Nutrients. 2020 Aug 8;12(8):2377. doi: 10.3390/nu12082377 (PMC7468958; doi:10.3390/nu12082377)
Supplement: Supplementary file 1 [file nutrients-12-02377-s001.zip › KW_Nutrients_SUPPLEMENTARY MATERIALS.docx]

**SUPPLEMENTARY MATERIALS**

**Supplementary Table 1. Odds ratio (ORs) of energy-adjusted CML for MOF and VFs in non-type 2 diabetics (non-T2DM), diabetics (T2DM), females and males in N=3949 with complete data on all covariates.**

| Fully adjusted  models | Major osteoporotic fractures (MOFs) | | | Vertebral fractures (VFs) | | |
| --- | --- | --- | --- | --- | --- | --- |
|  | Prevalence | ORs (95% CI) | p-value | Prevalence | ORs (95% CI) | p-value |
| Non T2DM | 9.4% | 1.08 (0.93-1.24) | 0.31 | 7.5% | 1.16 (1.01-1.33) | **0.03** |
| T2DM | 9.9% | 1.38 (0.97-1.98) | **0.08** | 7.4% | 1.10 (0.76-1.61) | 0.61 |
| Females | 11.6% | 1.10 (0.94-1.28) | 0.24 | 7.1% | 1.22 (1.02-1.45) | **0.03** |
| Males | 4.4% | 1.17 (0.91-1.49) | 0.22 | 8.0% | 1.11 (0.92-1.34) | 0.28 |

**Fully adjusted model: Energy-adjusted CML + age + sex + physical activity + dietary quality score + total energy intake per day+ eGFR + diabetes status + smoking status + BMI**

**Supplementary table 2. Odds ratio (ORs) of energy-adjusted total dAGE score (continuous) and quartiles (Q1-4), CEL and MG-H1 for MOFs and VFs in total population.**

| N = 3949 | Major osteoporotic fractures (MOFs) | | Vertebral fractures (VFs) | |
| --- | --- | --- | --- | --- |
|  | ORs (95% CI) | p-value | ORs (95% CI) | p-value |
| Energy-adjusted total dAGEs score | | | | |
| Total dAGE (cont’) | 1.07 (0.88-1.30) | 0.52 | 1.08 (0.89-1.31) | 0.43 |
| Q1 (ref.) |  |  |  |  |
| Q2 | 0.76 (0.54-1.07) | 0.53 | 0.82 (0.56-1.19) | 0.29 |
| Q3 | 0.89 (0.61-1.29) | 0.69 | 1.10 (0.74-1.63) | 0.64 |
| Q4 | 1.10 (0.69-1.76) | 0.28 | 1.38 (0.86-2.22) | 0.18 |
| Q1-3 vs. Q4 | 1.34 (0.94-1.90) | **0.11** | 1.46 (1.04-2.01) | **0.03** |
| Energy-adjusted MG-H1 | | | | |
| MG-H1 (cont’) | 1.00 (0.98-1.02) | 0.97 | 1.00 (0.98-1.01) | 0.73 |
| Q1 (ref.) |  |  |  |  |
| Q2 | 0.94 (0.67-1.31) | 0.72 | 0.96 (0.68-1.35) | 0.80 |
| Q3 | 0.91 (0.65-1.27) | 0.57 | 0.94 (0.67-1.32) | 0.71 |
| Q4 | 1.03 (0.73-1.45) | 0.88 | 0.98 (0.69-1.40) | 0.93 |
| Q1-3 vs. Q4 | 1.09 (0.82-1.45) | 0.58 | 1.03 (0.77-1.37) | 0.87 |
| Energy-adjusted CEL | | | | |
| CEL (cont’) | 1.00 (0.88-1.15) | 0.96 | 1.02 (0.90-1.17) | 0.73 |
| Q1 (ref.) |  |  |  |  |
| Q2 | 0.81 (0.58-1.13) | 0.22 | 1.12 (0.80-1.58) | 0.51 |
| Q3 | 1.00 (0.73-1.38) | 0.99 | 1.05 (0.75-1.49) | 0.77 |
| Q4 | 0.80 (0.57-1.12) | 0.20 | 1.06 (0.75-1.49) | 0.74 |
| Q1-3 vs. Q4 | 0.85 (0.64-1.14) | 0.28 | 1.00 (0.76-1.33) | 0.97 |

**Fully adjusted model: Energy-adjusted AGEs + age + sex + RS-cohorts + physical activity + dietary quality score + total energy intake per day+ eGFR + diabetes status + smoking status + BMI**
